# Evaluation of a spirituality informed e-mental health tool as an intervention for major depressive disorder in adolescents and young adults – a randomized controlled pilot trial

**DOI:** 10.1186/s12906-015-0968-x

**Published:** 2015-12-24

**Authors:** Badri Rickhi, Ania Kania-Richmond, Sabine Moritz, Jordan Cohen, Patricia Paccagnan, Charlotte Dennis, Mingfu Liu, Sonya Malhotra, Patricia Steele, John Toews

**Affiliations:** Canadian Institute of Natural and Integrative Medicine (CINIM), Calgary, AB Canada; Department of Psychiatry, Faculty of Medicine, University of Calgary, Calgary, AB Canada; Department of Obstetrics and Gynecology, Faculty of Medicine, University of Calgary, Calgary, AB Canada; Tom Baker Cancer Center, Calgary, AB Canada; Mental health Urgent Services, Foothills Hospital, Calgary, AB Canada; Child and Adolescent Addiction and Mental Health Programming NW Clinic, Calgary, AB Canada; Alberta Health Services, Calgary, AB Canada

## Abstract

**Background:**

Depression in adolescents and young adults is a major mental health condition that requires attention. Research suggests that approaches that include spiritual concepts and are delivered through an online platform are a potentially beneficial approach to treating/managing depression in this population. The purpose of this study was to evaluate the effectiveness of an 8-week online spirituality informed e-mental health intervention (the LEAP Project) on depression severity, and secondary outcomes of spiritual well-being and self-concept, in adolescents and young adults with major depressive disorder of mild to moderate severity.

**Methods:**

A parallel group, randomized, waitlist controlled, assessor-blinded clinical pilot trial was conducted in Calgary, Alberta, Canada. The sample of 62 participants with major depressive disorder (DSM-IV-TR) was defined by two age subgroups: adolescents (ages 13 to 18 years; *n* = 31) and young adults (ages 19 to 24 years; *n* = 31). Participants in each age subgroup were randomized into the study arm (intervention initiated upon enrolment) or the waitlist control arm (intervention initiated after an 8-week wait period). Comparisons were made between the study and waitlist control arms at week 8 (the point where study arm had completed the intervention and the waitlist control arm had not) and within each arm at four time points over 24-week follow-up period.

**Results:**

At baseline, there was no statistical difference between study and waitlist participants for both age subgroups for all three outcomes of interest. After the intervention, depression severity was significantly reduced; comparison across arms at week 8 and over time within each arm and both age subgroups. Spiritual well-being changes were not significant, with the exception of an improvement over time for the younger participants in the study arm (*p* = 0.01 at week 16 and *p* = 0.0305 at week 24). Self-concept improved significantly for younger participants immediately after the intervention (*p* = 0.045 comparison across arms at week 8; *p* = 0.0175 in the waitlist control arm) and over time in the study arm (*p* = 0.0025 at week 16). In the older participants, change was minimal, with the exception of a significant improvement in one of six factors (vulnerability) in study arm over time (*p* = 0.025 at week 24).

**Conclusions:**

The results of the LEAP Project pilot trial suggest that it is an effective, online intervention for youth ages 13 to 24 with mild to moderate major depressive disorder with various life situations and in a limited way on spiritual well-being and self-concept.

**Trial registration:**

ClinicalTrials.gov NCT00985686. Registered 24 September 2009.

## Background

Depression is a debilitating mental health condition gaining increasing attention given its impact on emotional, mental, and social processes. Worldwide, depression during adolescence is common, estimated to affect from 1 to 10 % [1–3] and up to 20 % [4, 5] of the population before the age of 18. In Canada, its prevalence is most common in those under 20 years of age [6–8], and unlike comparable countries, it has remained relatively unchanged in the adolescent and young adult populations [1]. Reasons for attention and concern regarding depression in the adolescent population are related to potentially negative consequences related to interruption in the development process, interpersonal conflicts, academic under-performance, low self-esteem, and suicide [2, 4, 5, 9]. Furthermore, development of adulthood depression is associated with youth onset [6, 10, 11].

In Canada, there is a wide range of youth mental health services, however most focus on a particular aspect (e.g. addiction, abuse), intervention/prevention programs and are often only available to those in crisis. Key youth services that address general distress and/or depression are challenged to meet the growing needs. Other barriers to access include transportation, particularly in rural areas [12], long wait times [13] as well as, youth’s reluctance to seek help due to perceived stigma [14, 15] inability to recognize a problem [16], and preference for self-reliance [17, 18]. Considering these limitations and the significant individual and societal burden of the condition, there is a critical need for development of treatment options that are effective and acceptable to this patient demographic.

Research in the area of depression and spirituality suggests that certain spiritual beliefs or experiences may play a beneficial role in the management of adolescent depression [19–21]. Forgiveness, spiritual support, daily spiritual exercises, hopefulness (or having faith), and self-ranking as religious or spiritual have been identified as specific factors which reduce depressive symptoms in adolescents [20]. Given these potential benefits, some authors have called for the inclusion of spiritual concepts in mental health interventions for youth [19, 22].

The term e-mental health refers to ‘mental health services and information delivered or enhanced through the internet and related technologies such as videoconferencing, web-based interventions, or social media, to name a few [23, 24]. E-mental health interventions are increasingly recognized as potentially useful and constructive mental health enhancing approaches [25], with potential applicability in the treatment and/or management of depression or depressive symptomatology [26, 27]. E-mental health has been identified as a key strategy for access to and provision of mental health services [24, 27]. Given the high use, access, and acceptance of the internet in youth [28], internet-based programming may be highly suitable to this population.

Existing online programs, however, tend to primarily focus on diagnosis and resource identification and do not directly engage youth. Those that do provide some help are generally workbook/DVD based and/or provided support through online/phone interaction with a therapy provider. It is important to consider that in an era where instant access is preferred and youth are not always comfortable discussing inner struggles with an adult stranger, although aimed at helping, these may also pose access barriers.

Although potentially beneficial, many of these online programs lack sufficient evidence [27] and are not readily available. In response to the need for appropriate and accessible services for depressed youth, the project team at the Canadian Institute of Natural and Integrative Medicine (CINIM) designed an online spirituality-informed intervention specifically targeting adolescents (13–18 years of age) and young adults (19–24 years of age). To our knowledge, this is the first clinical trial of an online spirituality-informed intervention for depression, specifically targeting adolescents and young adults.

### The LEAP project

The trial intervention was an eight-week online program called the LEAP Project (LEAP). It aims to treat and/or manage depression by empowering depressed youth with new perspectives and practical strategies to better manage life’s challenges. The label, LEAP, aims to capture the idea of leaping or moving forward in one’s life. This is achieved by guiding participants through an exploration of spiritually informed principles (e.g. forgiveness, gratitude, compassion) that are presented in eight modules (see Table 1). These principles were selected through an extensive literature review and were identified to be consistent across a wide range of spiritual practices and religious beliefs. The intervention is non-denominational and does not place emphasis on any one specific religious practice or tradition.

**Table 1:** Description of the eight LEAP project modules

*Module 1: Self-Acceptance (Breaking Through: Uncovering the REAL You).* Offers new ways to deal with negative thoughts and provides tools to help participants see themselves as they are.
*Module 2: Appreciation of Beauty and Creativity (Enjoying Again: Reconnecting with Life).* Explores ways to ease the feelings associated with depression, such as isolation and loneliness.
*Module 3: Mystery of Life (Coming Alive: Discovering Your Purpose).* Moves participants away from feelings of emptiness and boredom by showing them ways to connect with their passions.
*Module 4: Gratitude (Shifting Gears: Finding the Positive Spin).* Shows participants how to stop the downward spiral of “ruminating though patterns” by focusing on the positives.
*Module 5: Compassion and Giving (Reaching Out: Making a Difference*). Teaches participants how to handle the amplified feelings of helplessness and powerlessness that overwhelm them when they are depressed.
*Module 6: Acceptance (Moving On: Responding to Setbacks).* Provides participant with tools to deal with the inevitable challenges that life presents, despite the best planning.
*Module 7: Forgiveness (Breaking Free: Dealing with Past Hurts).* Presents ways to help participants let go of hurt, bitterness, and guilt when either they or someone else has done something they feel is unforgivable.
*Module 8: Celebration (Celebrating Possibilities: Moving Forward).* Encourages participants to plan events to celebrate their progress and enjoy the life ahead of them.

Participation required a weekly commitment of approximately two to three hours. The program length was chosen because psychotherapeutic depression interventions are commonly eight weeks in duration which appears to be sufficient to produce significant improvements [29]. The content is presented by a professional host who introduces and guides participants through the program materials. The host is an award winning writer, performer and producer. She was selected based on her ability to openly relate her own experience with clinical depression to the program content, contribution to script writing, and overall passion for the project.

LEAP takes an experiential and non-linear approach in order to facilitate independent and active participation rather than passive observation. The modules include fresh graphic designs with a multimedia format; video clips to illustrate the teaching content, including insights from a medical expert that have helped others move forward in their lives (Mastermind Sessions); music clips to validate teen’s emotions; youth autobiographical stories of personal struggle; off-line activities that allow participants to apply their newly obtained insights; relaxation techniques including downloadable visualizations; online journal and moderated comment boxes to share thoughts and experiences; extras section that includes humorous clips and jokes, and movie and book suggestions to reinforce the teachings; and links to resources such as depression information and support.

LEAP was created through a collaboration of researchers with content expertise at CINIM, the University of Calgary and Mount Royal University, and health professionals and educators from Alberta Health Services. To ensure its relatability and suitability to a youth audience, we also engaged twenty-five youth volunteers, aged 15 to 25, four of whom had previously experienced clinical depression. These volunteers attended focus groups and completed online assignments where they provided constructive input on all program content and design and suggested new program materials. Their feedback was used to further revise and refine the intervention.

This paper reports on the results of a waitlist controlled clinical pilot trial, which aimed to evaluate an innovative approach for managing depression in adolescents and young adults using a non-faith based spirituality program presented through an online platform. The research questions we aimed to address were:

1. Is the LEAP Project effective in reducing the severity of depression in adolescents and young adults diagnosed with major depressive disorder of mild to moderate severity?
2. Is the LEAP Project effective for improving secondary outcomes specific to spiritual well-being and self-concept in adolescents and young adults diagnosed with major depressive disorder of mild to moderate severity?

This paper presents the quantitative results. Findings from the qualitative data collected as part of the overall evaluation will be published separately, in a subsequent article.

## Methods

The LEAP Project was pilot tested using a parallel group, randomized, waitlist controlled, assessor-blinded clinical trial design. A waitlist design was considered to be appropriate given the lack of a suitable placebo or sham intervention. It is also a commonly used design in the evaluation of psychotherapeutic interventions for depression [30, 31]. In addition, offering the intervention to all participants reduces the potential of dropout in the control group.

The trial was conducted at CINIM, located in Calgary, Alberta, Canada. Ethics approval was obtained from the Conjoint Research Ethics Board at the University of Calgary (Ethics ID: E22549); Child Health Research Office, Alberta Health Services, (Ethics ID: 22549); Human Research Ethics Board, Mount Royal University (Ethics ID: 2011–39). The trial was also registered with ClinicalTrials.gov (Identifier: NCT00985686).

### Recruitment

All participants were recruited in Calgary, Alberta, Canada. Recruitment and all follow-up was completed between January 2010 and September 2012. Multiple recruitment strategies used included emails, presentations, mail outs, poster displays, local media (radio, television, print), and social media (Facebook). Local educational institutions (high schools, post-secondary), health professionals (physicians, psychologists, social workers, counsellors), and over 30 community organizations that provide services to youth were targeted. Neutral recruitment slogans were used (e.g. “Need help with depression? The LEAP Project might help”) in order to minimize potential selection bias in relation to the concept of spirituality.

Interested youth and/or their parents/guardians contacted the study coordinator by telephone and/or email. The coordinator explained the study details via telephone and confirmed the inclusion criteria (Table 2). If these criteria were met, youth were then screened using a two-step process. The initial screening was conducted by a registered nurse via telephone. The nurse asked questions to identify any exclusion criteria (Table 2). If no exclusion criteria were present, the potential participant was invited to attend an in-person screening with a physician and a registered nurse. The physician conducted a psychiatric assessment, which confirmed the diagnosis of major depressive disorder based on the Diagnostic and Statistical Manual for Psychiatry (DSM-IV-TR) and ruled out exclusion criteria (see Table 2). A registered nurse administered the age appropriate depression assessment scale (Children’s Depression Rating Scale Revised (CDRS-R) for 13–18 year olds or Hamilton Rating Scale for Depression (HAMD) for 19–24 year olds), which determined depression severity. Individuals 13 to 24 years of age were eligible if they met the DSM-IV-TR criteria for major depressive disorder (mild to moderate severity) and obtained a raw baseline score of 40 to 70 on the CDRS-R or 12 to 24 on the HAMD. In addition, study participation required agreement to committing two to three hours per week to complete each module and attending four to five in-person study visits.

**Table 2:** Protocol – eligibility criteria

Inclusion criteria:
• 13 to 24 years of age
• Suspicion he/she might be suffering from depression
• Stabilized on anti-depressants, if applicable
• Currently under the care of a health care professional
• Agreeable to having the study team contact the health professional prior to enrollment, at completion of study and if it was evident additional support was needed for the participant during the course of the study
• Interested in study participation
Exclusion criteria:
• High suicide risk
• History of multiple suicide attempts
• Recent death in the family
• History of Bipolar disorder, Psychotic disorder or Psychotic episodes
• Personality disorder traits that may impede participation in the study
• History of Attention Deficit Hyperactivity disorder (ADHD) (permitted if stabilized for at least 2 months on a long-acting medication, signs/symptoms/behaviours are well controlled, and participant agrees to continue)
• DSM-IV-TR diagnosis of substance dependence (except nicotine and caffeine) within the past 12-months
• Uncontrolled medical conditions in the last 3 months (assessed by qualified physician)
• Change in use of pharmacotherapy or herbal treatment for depression (St. John’s Wort) in the last 3 months OR during the first 2 months of trial participation (Eligible if no change in medication or dosage in the last 3 months and it is foreseeable that their current treatment will continue unchanged for the first 2 months of participation)
• Change in the use of medications that have mood altering effects in the last 3 months OR during the first 2 months of trial participation
• History of treatment resistance to ≥ 2 antidepressant medications when treated for an adequate period with a therapeutic dose
• Patients currently undergoing a specific psychotherapeutic treatment that has been shown to be effective for depression (such as Cognitive Behavioral Therapy (CBT) or Interpersonal Therapy (IPT)) or planning to start such therapy in the next two months

Individuals who did not meet study criteria, such as those whose depression severity exceeded study eligibility, and required additional support, were encouraged to follow-up with their health professional and given a list of community resources. The health professional was also notified by study staff of the ineligibility status. Study physicians were informed if individuals presented with potential safety risks during the screening process or after study enrolment and appropriate supports were obtained, including notifying the parents/guardians of all minor participants.

Consent was obtained from all participants prior to study participation; for participants under 18 years of age, informed assent and parental/guardian consent was required and obtained.

### Randomization and study groups

**Fig. 1:**
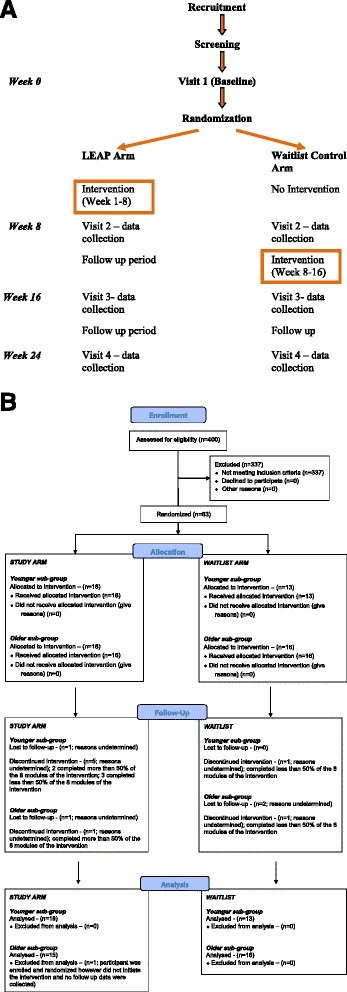
**a** Study Protocol. Schematic providing an overview of the study identifying recruitment, randomization, data collection points, and time points where intervention is implemented. **b** CONSORT Flow Chart. Standardized summary of enrolment, study group allocation, loss to follow-up and discontinuation of the intervention at follow-up, and participants included in the analysis

The study protocol is presented in Fig. 1a. Participants who met the eligibility criteria were randomized into one of two groups – the study arm or the waitlist control arm – using a 1:1 randomization ratio. The randomization list was generated by a statistician and maintained by an administrator who had no other involvement in the trial. The outcomes assessor was blinded to the participants’ allocation.

### The intervention

The intervention consisted of 8 modules, which were released on a weekly basis and were to be completed within the specific week. The study arm began the intervention immediately upon recruitment while the waitlist control arm commenced the intervention eight weeks after recruitment. Participants in both arms needed to be stable on their respective therapies and were instructed to maintain existing treatments for depression (e.g. pharmacotherapy, herbs, psychotherapy) during the eight-week intervention period. During the follow-up phase, there were no restrictions in the use of or change to any of the other depression treatments being used. Participants had optional access to the LEAP website during the follow-up phase.

### Data collection

Data collection took place between March 2010 and September 2012. Outcomes for depression, spiritual well-being, and self-concept were measured using standardized outcome measures with established psychometric properties and considered suitable for research purposes (see Table 3). Outcome measures differed for the younger and older participants; as such, the study sample consisted of two age subgroups: younger (13 to 18 years of age) and older (19 to 24 years of age). All outcomes were measured at four time points in both study groups. Baseline measures were completed at enrolment during visit 1. Data was then collected at week 8 (visit 2), when the study arm had completed the intervention; week 16 (visit 3) when the waitlist arm had completed the intervention; and, week 24 (visit 4) (see Fig. 1a). The depression severity measures were administered through semi-structured interviews. Inter-rater reliability between assessors was checked at four points during data collection was found to be high. All remaining outcome measures were self-administered.

**Table 3:** Outcome measures used to assess the LEAP project

Outcome	Younger group (13–18 years of age)		
	Instrument	Score Interpretation	Reference
Depression	Children’s Depression Rating Scale – Revised (CDRS –R)	↑ score = ↑ depression	Poznanski E & Mokros H, [47]
		A raw score of 40 or more is indicative of depression.	
Self-Concept	Piers Harris 2	↑ score = improved self-concept	Piers E & Herzberg D, [48]
		T-score ranges: < 29 is very low; 30–39 is low; 40–44 is low average, 45–55 is average; >56 is above average	
		Scores below 44 indicate low self-concept and 45–55 reflect average self-concept.	
Spiritual well being	Spiritual and Well-Being Scale (SWBS)	↑ score = ↑ spiritual well-being	Paloutzian R & Ellison C, [49]
		Scores of 40 or lower indicate low overall spiritual well-being.	
	Older group (19–24 years of age)		
	Instrument	Score Interpretation	References
Depression	Hamilton Depression Rating Scale (HAMD)	↑ score = ↑ depression	Williams et al., [50]
		Lower scores indicate improvement.	
		Scores of 12–19 indicate mild to moderate major depressive disorder.	
Self-Concept	6-Factor Rating Scale (SFSCS)	↑ score = improved self-concept	Stake L, 1994 [51] Yanico B & Lu T, [52]
Spiritual well-being	SIBS – Spiritual Involvement and Belief Scale	↑ score = ↑ spiritual well-being	Hatch et al., [53]

Program use was tracked by internal website statistics. Program completion was determined by login activity for all eight modules (see Table 5) within the eight-week time frame and clicking on all required components at least once (e.g. video and audio recordings). If required components were not viewed participants could not access the next module.

### Statistical analysis

A conservative “intention to treat” approach was used to guard against the potential for bias if dropouts are related to outcomes or group assignments, and preserve the original balance of random assignment. Average outcome measures by gender strata for the data collection points were used to replace missing values in the rare cases that outcome measures were not available for some subjects at the data collection points. As only 5 male participants were presented in the younger age subgroup, gender was not considered in stratification for the younger age subgroup.

Preliminary analyses were conducted to evaluate the influences of potential covariates, including gender, program completion status, and use of other treatments for depression (pharmacotherapy, psychotherapy or both) on outcome measures with general linear models. Covariates were evaluated by incremental F-test with *P* > 0.05 being considered non-influential, and therefore were not used as covariates in the final analyses. The final general linear models used to analyze the data included group as factor of interest to compare study vs waitlist arms. Gender and use of counselling were also included as covariates for statistical adjustments for the older age subgroup. Student *t*-test was used to test the separation of least squares means of study vs waitlist arms. Within group trend analysis was descriptive and was conducted using visit time as the only factor in the linear model to compare outcome measures across visit times. All analyses were conducted using SAS software (Version 9.3).

## Results

### Study sample and eligibility

In response to the recruitment efforts, 400 individuals inquired about study participation. Preliminary criteria were met by 196 individuals, who then participated in the telephone screening with a registered nurse. Of these, 92 were eligible for the screening visit with the physician and nurse, through which 63 were enrolled into the study.

Four participants were lost to follow-up. One participant was randomized, however, dropped out after baseline data was collected but before the intervention was initiated (reason undetermined). As such, the decision of the team was to remove this participant from the study, resulting in a final sample of 62 individuals. The sample consisted of two age subgroups: younger age subgroup (13 to 18 years of age; *n* = 31) and older age subgroup (19 to 24 years of age; *n* = 31). As this was a pilot trial, the study sample size allowed for reasonable treatment effects to be observed and calculated. Data was collected at baseline and three follow-up visits for all participants at 8, 16 and 24 weeks, with the exception of three participants: data was not collected at week 16 and 24 for one younger participant in the study arm and at 24 weeks for two older participants, both in the waitlist control arm. Reasons for loss to follow were not obtained. Baseline characteristics of the study sample are presented in Table 4.

**Table 4:** Baseline characteristics of study participants

	Study, Younger (*n* = 18)	Study, Older (*n* = 15)	Waitlist Control, Younger (*n* = 13)	Waitlist Control, Older (*n* = 16)
Characteristics				
Age - years (mean and range)	15.3 (12–18)	21.0 (19–24)	15.2 (13–17)	20.9 (19–24)
Gender				
Male	4	7	1	6
Female	14	8	12	10
Use of other treatments during intervention phase				
Anti-depressants only	3	4	2	1
Counselling only	4	5	3	4
Anti-depressants and Counselling	0	0	1	1
None	11	6	7	10
Education				
High School student	15	0	13	0
Post Secondary School	1	10	0	14
Not in school	2	5	0	2
Religious denomination indicated				
Yes	11	6	5	8
No	7	9	8	8
Work situation				
Working	5	8	3	10
Not working	13	7	10	6
Living situation				
With both parents	7	8	8	10
One parent	8	3	3	3
Partner	0	1	0	0
Roommate	0	2	0	0
Alone	0	1	0	1
Other	3	0	2	2

### Program completion

Of the 62 participants, 54 (87 %) competed the full eight-week LEAP project (see Table 5). Based on the age subgroups, 25 of 31 (81 %) of the younger participants and 29 of 31 (94 %) of the older participants completed the program. Of those who partially completed the program, three completed more than 50 % of the modules and five completed less than 50 % of the modules. Within the age subgroups, two (6 %) of the younger participants and one (3 %) older participants completed more than 50 % of the modules. Less than 50 % of the modules were completed by four (13 %) of the younger participants and one (3 %) older participant. Regardless of completion status, data collection at the four time points was completed for all 62 participants. An overview of the study results pertaining to enrolment and data collection is provided in Fig. 1b.

**Table 5:** Completion Rates of the Intervention (eight modules)

	Study Arm		
% of intervention modules completed	Younger subgroup (*n* = 18)	Older subgroup (*n* = 15)	TOTAL
100 %	13	14	27
99-51 %	2	1	3
50 % or less	3	0	3
	Waitlist Arm		
% of intervention modules completed	Younger subgroup (*n* = 13)	Older subgroup (*n* = 16)	TOTAL
100 %	12	15	27
99-51 %	0	0	0
50 % or less	1	1	2

### Outcomes

The impact of the intervention was based on the primary outcome of depression severity and secondary outcomes of spiritual well-being and self-concept. P-values presented (incremental F-test with P > 0.05 indicating non-significance) were adjusted for gender in the older sub-group and counselling in both age groups. At baseline, there was no significant difference in the three outcomes across the study groups (study vs waitlist control) in either age subgroup.

### Depression severity

#### Younger age subgroup

**Fig. 2:**
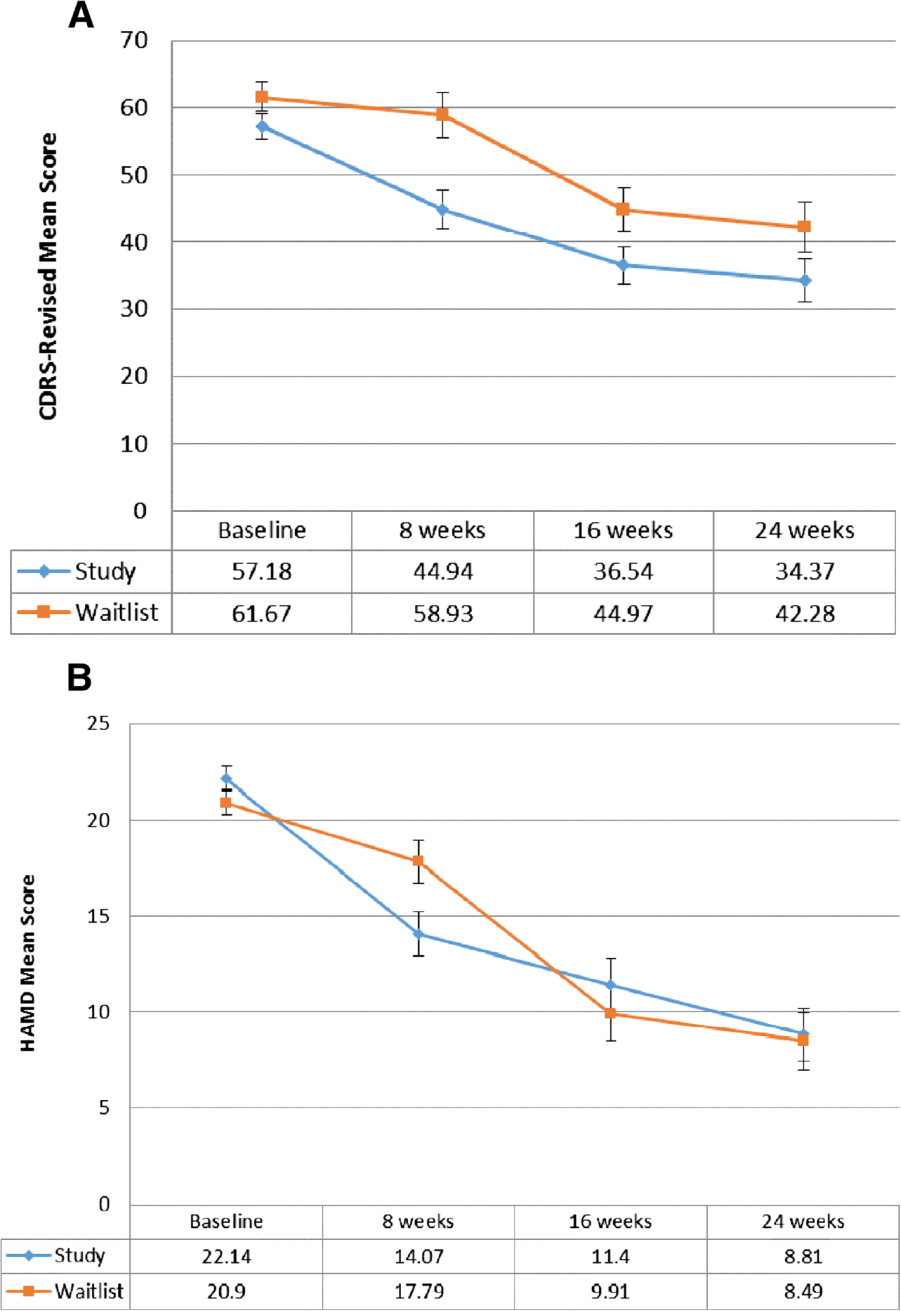
**a** Depression Severity - CDRS-Revised Mean Scores (Younger subgroup). Results of the statistical analysis comparing mean scores within each study group across time and across study groups at week 8 are presented in the text of the results section. Level of significance: *p* ≤ 0.05. **b** Depression Severity - HAMD Mean Scores (Older subgroup). Results of the statistical analysis comparing mean scores within each study group across time and across study groups at week 8 are presented in the text of the results section. Level of significance: *p* ≤ 0.05

For the study arm, the mean CDRS-R score decreased significantly (*p* = 0.035) from 57.18 (SE = 1.87) to 44.94 (SE = 2.86) after the intervention at week 8 (see Fig. 2a). Over the follow-up period, depression severity continued to improve, with a significant decrease (*p* = 0.0001) to 36.54 (SE = 2.77) at week 16 and to 34.37 (SE = 3.22) at week 24. For the waitlist control arm, the mean CDRS-R score decreased from 61.67 (SE = 2.21) at baseline to 58.93 (SE = 3.37) at week 8, however, this difference was not significant (*p* = 0.4799). After the intervention (week 8), the score significantly decreased (*p* = 0.0017) to 44.97 (SE = 3.26) at 16 weeks, and significantly decreased further (*p* = 0.002) at 24 weeks to 42.28 (SE = 3.79) compared to the score of 58.93 at 8 weeks.

Comparing across the study groups (study vs waitlist control), there was no difference in the severity of depression (*p* = 0.133) at baseline. At week 8, the decrease in depression severity of the study arm (MS = 44.94, SE = 2.86) was significantly greater (*p* = 0.0038) compared to the waitlist control arm (MS = 58.93, SE = 3.37).

#### Older age subgroup

For the study arm, the mean HAMD score significantly decreased (*p* = 0.0001) from 22.14 (SE = 0.65) at baseline to 14.07 (SE = 1.13) at week 8 (see Fig. 2b). The decrease continued to be significant (*p* = 0.0001) at 16 weeks (MS = 11.4, SE = 1.39) and at 24 weeks (MS = 8.81, SE = 1.4). For the waitlist control arm, the decrease in the mean HAMD score between baseline (MS = 20.90, SE = 0.65) and 8 weeks (MS = 17.79, SE = 1.12) was not significant. After the intervention, the decrease in mean scores from 17.79 (SE = 1.12) at week 8 to 9.91 (SE = 1.39) at week 16 was significant (*p* = 0.0001), and to 8.49 (SE = 1.49) at week 24 (*p* = 0.0001).

As with the younger subgroup, at baseline, there was no significant difference (*p* = 0.1844) in the severity of depression across the two study groups (study vs waitlist control). At week 8, there was a significant difference (*p* = 0.0244) in the severity of depression in the study arm (MS = 14.07, SE = 1.13) compared to the waitlist control arm (MS = 17.79, SE = 1.12).

### Spiritual well-being

#### Younger age subgroup

**Fig. 3:**
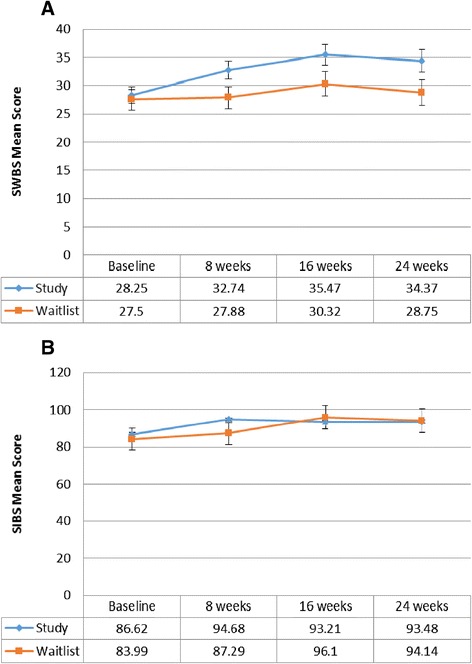
**a** Spiritual Well-Being – SWBS Mean Scores (Younger subgroup). Results of the statistical analysis comparing mean scores within each study group across time and across study groups at week 8 are presented in the text of the results section. Level of significance: *p* ≤ 0.05. **b** Spiritual Well-Being - SIBS Means Scores (Older subgroup). Results of the statistical analysis comparing mean scores within each study group across time and across study groups at week 8 are presented in the text of the results section. Level of significance: *p* ≤ 0.05

In the study arm, the SWBS mean scores did not significantly change (*p* = 0.1096) from 28.25 (SE = 1.50) at baseline to 32.74 (SE = 1.65) at week 8 (see Fig. 3a). Compared to the baseline measure, the increase to 35.47 (SE = 1.82) at 16 weeks was significant (*p* = 0.0101) and to 34.37 (SE = 1.94) at 24 weeks (*p* = 0.0305). For the waitlist control arm, there was no change in the mean scores between baseline (MS = 27.5, SE = 1.77) and week 8 (MS = 27.88, SE = 1.94). The minimal changes in the mean scores between week 8 (MS = 27.88, SE = 1.94), and week 16 (MS = 30.32, SE = 2.14) and week 24 (MS = 28.75, SE = 2.29) were not significant (*p* > 0.10).

Comparing across arms (study vs waitlist control), at baseline, there was no difference in the level of spiritual well-being (*p* = 0.7493). At week 8, spiritual well-being in the study arm (MS = 32.74, SE = 1.65) was approaching a significant difference (*p* = 0.0663) compared to the waitlist control arm (MS = 27.88, SE = 1.94). The improvement in the study arm compared to the waitlist control arm was also approaching a significant difference at week 16 (*p* = 0.0784) and week 24 (*p* = 0.072).

#### Older age subgroup

Although a trend towards improvement in spiritual well-being within the study arm is suggested by the increase in SIBS mean scores from 86.62 (SE = 5.81) at baseline to 94.68 (SE = 5.8) at week 8 and within the waitlist control arm from 87.29 (SE = 5.79) at week 8 to 96.1 (SE = 6.33) at week 16, the difference was not significant (*p* > 0.10) in either of the study groups (see Fig. 3b). The level of spiritual well-being across the study groups was not significantly different at baseline or following the intervention and week 8, 16, and 24 (*p* > 0.10).

### Self-concept

#### Younger age subgroup

**Fig. 4:**
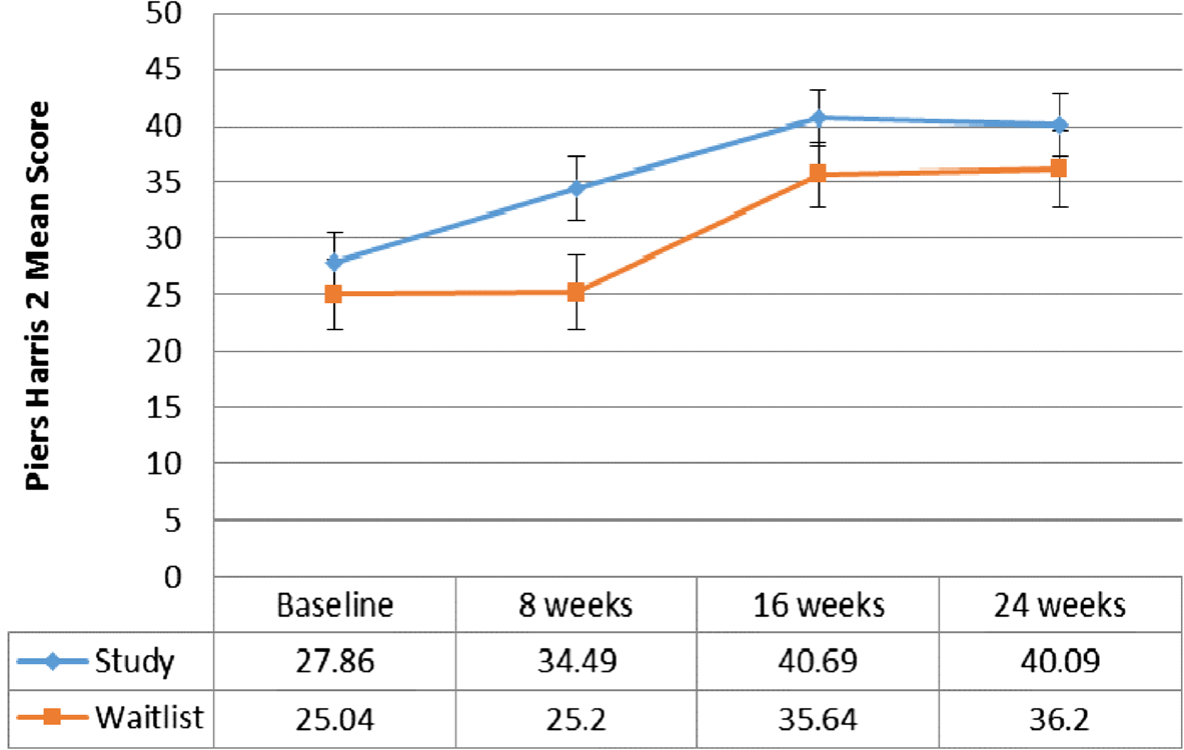
Self-Concept - Piers Harris 2 Mean Scores (Younger subgroup). Results of the statistical analysis comparing mean scores within each study group across time and across study groups at week 8 are presented in the text of the results section. Level of significance was set at *p* **≤** 0.05

For the study arm, the increase in Piers Harris 2 mean scores from 27.86 (SE = 2.69) at baseline to 34.49 (SE = 2.79) at week 8 was not significant (*p* = 0.1311). At week 16, the improvement in self-concept (MS = 40.69, SE = 2.5) was significantly different (*p* = 0.0025) from baseline, plateauing during the follow-up period (see Fig. 4). For the waitlist control arm, compared to the mean score of 25.20 (SE = 3.29) at 8 weeks, the improvement was significant (*p* = 0.0175) after the intervention at 16 weeks (MS = 35.64, SE = 2.94) and approaching significance (*p* = 0.096) at 24 weeks (MS = 36.2, SE = 3.41).

Comparing study arm to the waitlist control arm, mean scores reflecting self-concept were not significantly different (*p* = 0.5027) at baseline. At week 8, the study arm demonstrated a significant improvement (*p* = 0.0405), with a mean score of 34.49 (SE = 2.79) compared to 25.20 (SE = 3.29) in the waitlist control arm. At weeks 16 and 24, mean scores plateaued in both arms and the differences were not significant across the arms.

#### Older age subgroup

Of the six factors used to measure self-concept, improvement in task accomplishment (T-factor) was approaching a significant difference (*p* = 0.0775) for the waitlist control arm at 24 weeks (MS = 30.22, SE = 1.45) compared to week 8 (MS = 26.36, SE = 1.42). A decrease in vulnerability (V-factor) in the study arm was significant (*p* = 0.025) at 24 weeks (MS = 24.13, SE = 1.78) compared to baseline (MS = 29.56, SE = 1.39). A decrease in vulnerability was also approaching significance (*p* = 0.0671) in the waitlist control arm at 24 weeks (MS = 24.24, SE = 1.77) compared to 8 weeks (MS = 29.19, SE = 1.62).

At baseline, the mean scores of the six factors did not differ significantly across the study groups (study vs waitlist control). At week 8, there was no significant difference across the study groups, with the exception of giftedness (G-factor), where the improvement in the study arm (MS = 22.23, SE = 1.68) compared to the waitlist control arm (MS = 17.96, 1.68) was approaching a significant difference (*p* = 0.0787).

## Discussion

E-mental health is rapidly developing and it is anticipated that it may have a profound effect on the way in which people access health care and health care information [27, 32]. However, there is a lack of high quality empirical evidence to support the effectiveness and overall benefit of e- mental health programs. In a recent review of e-mental health programs, Christensen and Petrie (2013) [27] report that of the 15 depression specific programs identified internationally, around half were supported by any evidence and only one third were examined through a randomized controlled trial. To our knowledge, the results presented here are the first rigorous evaluation of a Canadian spirituality-informed e-mental health intervention targeting depressed youth.

The results of this study indicate that the LEAP Project is effective in significantly decreasing depression severity in adolescents and young adults diagnosed with major depressive disorder of mild to moderate severity. The reduced severity was also maintained after the intervention was completed during the follow-up period, suggesting the potential longer term impact of what was learned through the LEAP modules. For the younger participants, LEAP also had a positive effect on spiritual well-being and self-concept.

The effect of the intervention on reducing depression severity in both the younger and older participants is also considered to be clinically significant. A treatment response using the CDRS-R has been defined as a reduction in mean scores 30 % to 50 % from baseline [31, 32]. For the younger participants, the response was over 30 % in the study group during the follow-up period (36 % at 16 weeks and 40 % at 24 weeks). Similarly, in the waitlist group, severity decreased by 27 % (16 weeks) and 31 % (24 weeks). It is important to emphasize that the decrease was not short term; on-going effects are observed weeks after the intervention had been completed, suggesting the potential long term benefits of this intervention. Further, in the study group, at weeks 16 and 24, the CDRS-R mean score was below 40, indicating that the change is not only a reduction in depression severity but a potential shift towards alleviation of depressive symptomology altogether [4, 33]. In the waitlist group, a similar trend is apparent, however, scores hover around 40. This may be related to the fact that the follow-up period after the intervention in the waitlist control group was shorter. A decrease in scores below 40 may have been observed if follow-up had taken place at 32 weeks. In the older age sub-group, the decrease in depression severity in both the study and waitlist control groups at 16 and 24 weeks ranged from 49 % to 60 %, which is recognized as a clinically significant change in the depression severity [34]. In addition, in both arms, the difference in mean scores from baseline to each of the follow-up points differed by more than 3 points, which is a common measure of clinically significant change based on mean scores [35, 36].

The improvement in self-perceived spiritual well-being of the younger participants was approaching statistical significance (*p* < 0.10), however it is important to note that the interpretation of the mean scores indicates that the change was from “very low” to “low” spiritual well-being. Therefore, this may not be considered a clinically meaningful improvement. However, such a finding is acceptable, and to some degree even expected. Several models of spiritual development [37–39] identify it as progressive, evolutionary process during adolescence that is marked by reflection and is closely linked to identity formation. As such, the modest improvement is important; it suggests that exposure to the concepts and ideas presented had some positive effect. The continued improvement observed at 24 weeks in the study arm is also indicative of the fact that integration of the concepts may occur over time and continue after exposure.

Psychological development during adolescence consists of many overlapping tasks. These include the development of emotional and psychological independence that ends with a stable identity formation or conceptualization of self. In his developmental theory, Erik Erikson [40] discussed how we need to negotiate psychosocial crises at each stage of development. Identity formation versus role confusion is this crisis that occurs during the teenage years. The spirituality-informed modules contained many topics that led participants to ask key questions that contribute to identity formation (e.g. who am I? what is my purpose?). This may speak to the influential role this online spirituality-informed tool played in helping youth explore and develop their concept of self. In the case of our older participants, it is plausible they may have already gone through the stage of identity formation and, thus, this intervention had less of an effect on improving thoughts related to who they are, how they relate to others and their purpose.

Spirituality is increasingly recognized as an important consideration in mental health and mental health interventions, yet we found that the use of the term in labelling or describing the LEAP Project problematic in several ways. First, spirituality as a concept is quite difficult to define in a cohesive way that is acceptable to different groups of stakeholders. Although we drew a distinction between spirituality and religion, to the lay public, the two concepts are closely connected and often used synonymously. Second, there is a range of personal perceptions, negative and positive, attached to the concept of spirituality, which then impacted how LEAP is received. For example, inclusion of the term in a descriptive label resulted in recruitment challenges, where we were unable to recruit at one school district, parents of potential participants expressed apprehension/caution, and questions were raised as to whether or not LEAP would challenge one’s existing belief systems or religious values. To overcome this challenge, we created guest accounts so that the program could be previewed. In addition, a significant amount of time was also committed to responding to concerns, explaining the neutral orientation of LEAP in context of religious beliefs, and attention was drawn to how the intervention explored foundational life principles. These efforts led to building relationships with potential referral sources, participants and parents, which helped to establish credibility of the program and facilitated recruitment.

There are several limitations of the study that require attention. The early stage of this evaluation warranted a pilot study with a design that precluded the inclusion of a comparison placebo arm. We also opted to use a waitlist trial design given its advantages [30, 41], particularly in relation to psychotherapeutic interventions. However, it is important to recognize that concerns have been raised in the research community regarding the potential for over estimation of intervention effects using such a design [42, 43]. The explanation provided for this possible outcome is that participants in the waitlist control arm improve less than controls in non-waitlist designs as there is an expectation they are to “wait to change” until they receive the intervention [44]. In our study, it is expected that such an effect would be minimal given that participants in our waitlist control arm were not restricted from continuing existing care or treatment for their depression during the wait period. As such, it is anticipated this supported a mind-set of continued effort for self-improvement. An additional design related issue is that of blinding. Although assessor blinding was achieved, participants were not blinded to the intervention. Given the nature of the intervention and study design, a suitable control group was not possible. This may bias the results, particularly given that outcomes are based on patient self-reports. We aimed to present LEAP in a neutral manner in relation to the concept of spirituality; however, it cannot be ruled out that participants with an open or accepting attitude towards spirituality (and related concepts) may have self-selected for participation, thus creating a potential for selection bias. The use of standardized outcome measures resulted in the need to create two age sub-samples, reducing the sample size (each group to n = 31). This limited our ability to conduct a stratified analysis to identify potential confounders to our findings. However, the small size of the groups was sufficient for the purposes of this pilot trial. Lastly, seasonal effects, which may have had an impact on observed outcomes, were not controlled for in this study.

As e-mental health interventions are embraced and likely to continue developing, there are several areas that require attention in future evaluations of such programs. The amount of time spent on a website or specific online module (or the dosing effect) is an important factor which needs to be considered in association to the observed outcomes. The key issue around dosing is to be able to assess whether it is any or a certain level of exposure that has an effect. Although e-mental health programs may provide opportunity for greater access to services, the digital nature of the intervention may also present its own barriers. Jorm et al., (2013) [45] points out that access to the internet or devices (e.g. smart phones) is not necessarily equal for all individuals (at the local, national and international levels). There is also some evidence suggesting that most users of e-interventions are those who have access to other services and are health literate [46]. Future evaluations of such approaches should therefore include data collection on specific participant characteristics such as the level of health literacy and types of services that have or are being accessed by the individuals opting to use or sign up for e-mental health programs.

## Conclusions

The LEAP Project pilot trial makes an important contribution to our knowledge about the potential effectiveness of spiritually-informed e-mental health interventions for mild to moderate major depressive disorder in adolescents and young adults. The good compliance supports the possibility that the program could be successfully implemented in a community setting. The LEAP Project also presents a promising tool that addresses many of the barriers faced by individuals seeking resources to address mental health problems or issues, including location, access, and stigma. Given that the majority of youth use the internet and the average Canadian spends over 40 h online per month, the program’s online delivery method may help overcome some of the challenges accessing mental health care. The findings of this pilot study also illustrate how the internet can be harnessed to positively impact the mental health of adolescents and young adults.
